# Aberrant Methylation of APAF-1 Gene in Acute Myeloid Leukemia Patients

**Published:** 2017-07-01

**Authors:** Shahrbano Rostami, Fatemeh Nadali, Reza Alibakhshi, Farhad Zaker, Nahid Nasiri, Mehrdad Payandeh, Bahram Chahardouli, Ali Maleki

**Affiliations:** 1 Hematology-Oncology and Stem Cell Transplantation Research Center, Tehran University of Medical Sciences, Tehran, Iran; 2 Department of Hematology, School of Allied Medical Sciences, Tehran University of Medical Sciences, Tehran, Iran; 3 Department of Biochemistry, School of Medicine, Kermanshah University of Medical Sciences, Kermanshah, Iran; 4 Cellular and Molecular Research Center, Iran University of Medical Sciences, Tehran, Iran; 5 Department of Hematology, School of Allied Medical Sciences, Iran University of Medical Sciences, Tehran, Iran; 6 Department of Hematology, Oncology, Kermanshah University of Medical Sciences, Kermanshah, Iran

**Keywords:** Acute myeloid leukemia, Epigenetics, Methylation, APAF-1, MSP

## Abstract

**Background:** Acute myeloid leukemia (AML) is a heterogeneous clonal disorder characterized by immature myeloid cell proliferation and bone marrow failure. Various genetic and epigenetic factors have been found to be influential in such patients.

Methylation silencing of APAF-1, a putative tumor suppressor gene (TSG), has been found in several human malignancies. In this study, we explored the association of APAF-1 methylation status with AML patients.

**Materials and Methods:** We studied the methylation status of APAF-1 gene in 101 AML patients and 50 healthy subjects as controls. Genomic DNA was extracted from leukocytes in peripheral blood or bone marrow and the methylation status of APAF-1 gene promoter was detectedusing methylation-specific PCR (MSP) method with specific methylated and unmethylated primers. Gene expression was analyzed using real time RT-PCR.

**Results: **The prevalence of methylated (MM) and hemi-methylated (MU) CpG dinucleotides within the APAF-1 gene promoter of AML patients was 12 (11.9%) and 45 (44.6%), respectively, while no methylation was detected in the control samples (p < 0.001). Our results showed a higher frequency of methylated APAF1 in FLT3-ITD mutated cases (p=0.04). APAF1 mRNA expression was significantly lower in methylated cases compared with normal cases.

**Conclusion:** The present study indicated the increased frequency of hypermethylation of APAF-1 gene promoter in AML patients. APAF-1 aberrant CpG island methylation was associated with transcriptional downregulation in AML patients. Therefore, promoter methylation of APAF-1 gene could be considered as an epigenetic factor that contributes to the development of AML.

## Introduction

 Acute myeloid leukemia (AML) is a form of blood cancer that is characterized by infiltration of the bone marrow, blood, and other tissues by proliferative, clonal, abnormally differentiated, and occasionally poorly differentiated cells of the hematopoietic cells^   1 ^. AML is the most common acute leukemia in adults and the second most frequent leukemia among children^   2 ^. It is now cured in 35-40% of adult patients who are 60 years of age or younger and in 5-15% of patients older than 60 years^   1 ^. 

The development of AML is associated with the accumulation of acquired genetic alterations and epigenetic changes in hematopoietic progenitor cells, which alters the normal mechanism of cell growth, proliferation, death, and differentiation^   3 ^. Significant progress in the diagnosis of molecular abnormalities has revealed that approximately 86% of AML patients have at least one defect in their molecular structure^   4 ^. NPM1 and FLT3 are the most important genes that have already been evaluated in patients with AML^   5 ^. In addition, numerous other genes have been identified that contribute to epigenetic modifications^   6 ^. The promoter methylation of specific tumor suppressor genes is a mechanism for the multi-step model of tumorigenesis. If methylation occurs within the promoter region of a suppressor gene, epigenetic silencing of this gene may lead to functional inactivation, a mechanism reported for various tumor entities^   7 ^.

It has been shown that the deregulation of apoptosis results in irregular cell survival, which has been implicated in the development of cancer. Apoptotic protease activating factor 1 (APAF-1), a putative tumor suppressor gene (TSGs), encodes one of the important cytoplasmic proteins in DNA damage-induced apoptosis and is therefore essential for tumor suppression ^8^^,^^9^ .

APAF-1 forms apoptosome along with cytochrome c and adenine nucleotides and then recruits and activates caspase-9, which in turn activates the executioner caspases, including caspase-3 and -7. The active executioners kill the cell by proteolysis of key cellular substrates^   10 ^. 

Since the methylation of APAF-1 may play a role in the initiation and leukemogenesis of AML, in the present study, we investigated the methylation status of this gene in CpG islands of its promoter in newly diagnosed AML patients. In addition, the association between methylation status of APAF-1and its expression with clinicopathological features of patients was also evaluated.

## MATERIALS AND METHODS

 This study was approved by the Ethics Committee of TUMS. Informed consent was obtained from individual participant included in the study. Peripheral blood samples were drawn from 50 healthy individuals (negative control group) and 101 patients with newly diagnosed AML who were admitted to the Hematology, Oncology and stem cell Transplantation Research Center, Shariati Hospital. All patients were classified according to the FAB classification. The initial demographic data and clinical parameters, including white blood cell count, platelet count, age, and hemoglobin were extracted from patients' medical records. 

**Genomic DNA extraction**

DNA of patients and controls was extracted from EDTA-treated whole blood. From each subject, 5 ml peripheral blood sample was collected into tubes containing EDTA. The mononuclear cells, including leukemic blast cells were isolated by sedimentation on Ficoll-Paque Plus (GE Healthcare Life Sciences, Uppsala, Sweden) density gradients. Then, genomic DNA was extracted using standard salting-out method.

**Detection of FLT3-ITD and NPM1 mutation**

FLT3-ITD and NPM1 mutations were determined using multiplex PCR Fragment length analysis method as previously reported^   11 ^**.**

**Bisulfite conversion**

The extracted DNA underwent bisulfite conversion (EpiTect Bisulfite kit, QIAGEN, USA), which is based on the chemical conversion of unmethylated cytosine to uracil where methylated cytosine remains intact. Modified DNA was suspended in elution buffer and was immediately used or stored at -80 °C.

**Methylation-Specific PCR (MSP)**

APAF-1 promoter methylation status was detected using MSP method, which has facilitated the detection of methylation status at CpG islands in cell lines as well as biological and clinical samples^   12 ^. DNA-treated samples were subjected to polymerase chain reaction (PCR), which was performed with two primer sets of methylated (M) and unmethylated sequence (U). This approach could detect homozygous (MM) or heterozygous (MU) methylation status in the samples. We used two primer pairs specified for checking the methylated or unmethylated residues. These primers are shown in Table 1. The sequences of these primers were designed by MethPrimer software (http://itsa.ucsf.edu/~urolab/methprimer)^   13 ^.

**Table 1:** Specific primer Sequences were used for the study of APAF-1 promoter methylation

**Gene name**	**M/U**	**Primer set (5′–3′)**		
		**Forward**	**Reverse**	
APAF-1	M	TATTGCGATATTGTTTTAAATTCGA	GAAACGTAACTAAACCTCAAAAACG	
	U	TATTGTGATATTGTTTTAAATTTGA	CAAAACATAACTAAACCTCAAAAACAC	

MSP-PCR was performed in 25 𝜇L volume containing 20 pM of each forward and reverse primers, 2 uL of treated DNA, 200 uM of dNTPs, 1.5 mM of MgCl2, 1 U of Taq polymerase, and 2.5 uL of 10X PCR buffer. After denaturing at 94°C for 5 minutes, the amplification was conducted with 35 cycles at 94°C for 45 seconds, 53°C for 45 seconds, and 72°C for 45 seconds, followed by re-extension for 10 min at 72°C. The PCR products were loaded onto 1.5% agarose gel containing GelRed and visualized under UV gel documentation (Quantum ST4). 

**Figure 1:**
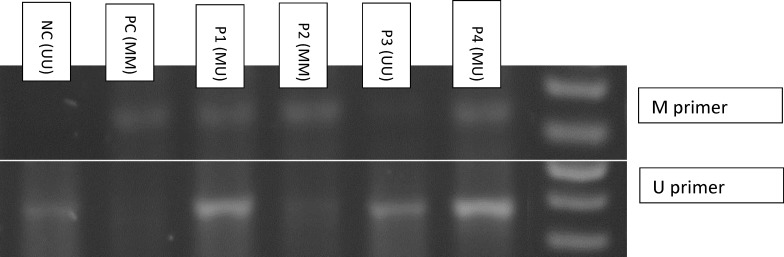
Agarose gel electrophoresis pattern of MSP products of the APAF-1 gene in controls and four AML Patients. NC= nonmethylated control, MC= methylated control , P= Patient. Patient 1 shows APAF-1 hemimethylated (MU) state, Patient 2 demonstrates methylated (MM) state, Patient 3 shows unmethylated (MU) state and Patient 4 indicate the hemimethylated (MU) state of APAF-1 gene.

In this research, EpiTect PCR control DNA kit (Qiagen, Valencia, CA), including unmethylated and completely methylated DNAs as negative and positive controls were used. Meanwhile, ddH2O served as a blank control. Agarose gel electrophoretic pattern of some MSP products of APAF-1 gene is shown in Figure 1. 

**Gene expression analysis**

The expression level of APAF-1 was evaluated by quantitative RT-PCR. RNA was isolated from the mononuclear cells (MNCs) by TRIzol® (Invitrogen, Carlsbad, CA) according to the manufacturer's instructions. cDNA was prepared using the Prime Script RT reagent kit (*Takara*Bio, Inc., Otsu, Japan) following manufacturer’s guidelines. qPCR was conducted using the real-time PCR kit (Takara Bio Inc. Otsu, Japan) on Rotor Gene 6000 system. β-actin was simultaneously applied as the housekeeping gene to normalize the expression of 

genes. The cycling conditions were as follows: 30 sec at 95°C, 40 cycles for 5 sec at 95°C and 30 sec at 60°C (combined annealing/extension step). Each reaction was done in triplicate. After gene amplification, the melting curve analysis was performed. 

The relative mRNA expression level of samples was calculated using the ∆∆Ct method as well as comparison with the amount of mRNA in control samples. The primers related to APAF-1 (Hs_APAF-1_1_SG QuantiTect Primer Assay [NM_001160]) and beta actin (Hs_ACTB_1_SG QuantiTect Primer Assay [NM_001101]) used in this study were purchased from QIAGEN Company (Valencia, CA).

**Statistical analysis**

Chi-square and Mann-Whitney U test test were used to analyze the association between promoter methyaltion status of APAF1 and demographic and molecular characteristics. p<0.05 was considered statistically significant. All data were analyzed using SPSS version 19.0 software (Statistical Package for Social Service Inc., Chicago, IL, USA).

## Results

 Demographic, biochemical, and molecular characteristics of the study population are summarized in Table 2.

**Table2:** Demographic and molecular characteristics of patients.

**Characteristics **	**Cases** **n=101**
Gender Male Female	60(59.4) 41(40.6%)
Age in years mean (range)	41.5(18-75)
WBC(109/L) mean (range)	32(700-430)
Hb (g/dl) mean (range)	9(4.9-15)
Plt ( 109/L) mean (range)	77.6(2-691)
FLT3-ITD mutation(Positive/negative)	29/72
NPM1 mutation(positive/negative)	12/89

A total of 101 patients with newly diagnosed AML entered the study. There were 60 males (59.4%) and 41 females (40.6%) with a median age of 41.5 years (range 18-75). The highest incidence of AML was observed in the age category of 30-39 years (35.6%), followed by 18-29 (28.7%) and 49-40 (22.8%) years, respectively. AML-M2 (36.6%) as well as AML-M4 (20.8%) and AML-M1 (13.9%) were the most frequent subtypes in this series.

Molecular evaluation of the patients has shown that 28.7% and 11.9% of the studied individuals were positive for NPM1 and FLT3 mutations, respectively (Table 2). Distribution of APAF-1 promoter methylation status is shown in Table 3. The prevalence of methylated (MM) and hemi-methylated (MU) CpG dinucleotides within the APAF-1 gene promoter of AML patients was 12 (11.9%) and 45 (44.6%), respectively, while no methylation was detected in the control samples (p < 0.0001). Forty- four patients (43.6%) were ummethylated. 

In further analysis, cases which showed a visible band of methylated PCR product were considered as methylated.

**Table 3:** APAF-1 promoter methylation status in cases

**Methylation status**	**Patients(N=101)**
Methylated(MM)	12(11.9%)
Hemimethylated(MU)	45(44.6%)
Unmethylated(UU)	44(43.6%)
MM+MU	57(56.4%)

Our analysis showed a higher frequency of APAF1 promoter methylation in FLT3-ITD mutated cases (22/29) compared with FLT3-wildtype (WT) patients (38/72) (p=0.04).

There were no significant differences in hematological parameters between patients with and without APAF1 methylation, except for WBC count (p=0.06).Moreover, the distribution of APAF-1 promoter methylation status showed no significant difference in various age groups. The findings of this study showed a trend of sex difference (higher in females, Table 4).

**Table 4:** APAF1 methylation status by demographic and molecular features

**Factors**	**Unmethylated**	**Methylated**	**P-Value**
Sex Male Female	29 (28.7%) 12(11.8%)	31(30.8%) 29(28.7%)	p =0.065
Age(years) 18-59 ≥60	38(37.6%) 3(2.97%)	54(53.4%) 6(5.95%)	p=0.7
FLT3-ITD mutation Positive Negative	7(6.93%) 34(37.66%)	22(21.78%) 38(37.62%)	p=0.04
NPM1 mutation Positive Negative	2(1.98%) 39(38.61%)	10(9.9%) 50(49.5%)	p= 0.1
Plt ( 109/L) mean (range)	100.2(4-61.9)	61.9(2-270)	p=0.55
WBC(109/L) mean (range)	23.25(0.8- 187.7)	39.12(0.7- 430)	p=0.06
Hb (g/dl) mean (range)	9.4(4.9-61.9)	8.7(5.3-14.8)	p=0.14

The distribution of APAF-1 promoter methylation was also compared in various subgroups of AML and no significant differences were observed among the studied patients (Table 5). In real*-*time quantitative RT-PCR, we found that APAF-1promoter methylation was inversely correlated with mRNA expression in patients. There was a significant difference between the mean delta CT value of the controls compared to the mean delta CT of patients with methylated APAF1 using the two-tailed t-test (P= 0.009).

**Table5:** The distribution of APAF-1 methylation status in different AML subtype

**AML Subtype**	**Unmethylated**	**Methylated/ ** **Hemimethylated**	**P-Value**
M0	2(1.98%)	1(0.99%)	p=0.3
M1	2(1.98%)	12(11.88%)	
M2	18(17.82%)	20(19.8%)	
M3	4(3.98%)	5(4.95)	
M4	8(7.98%)	13(25%)	
M5	2(1.98%)	6(5.94%)	
M6	2(1.98%)	2(1.98%)	
M7	1(0.99%)	0	
AML/MDS	2(1.98%)	1(0.99%)	

## Discussion

 Apoptosis, the programmed cell death, is highly important in the development and homeostasis of the hematopoietic system. Any deregulation of apoptosis is involved in the development of a large number of human diseases, including cancer^   3 ^. Defective apoptosome proteins (e.g. APAF-1) have been implicated in a number of human malignancies, including AML^   3 ^.

APAF-1 (Apoptosis Protease Activating Factor-1) is important for tumor suppression and is a central component of the intrinsic pathway of apoptosis, which is vital for cellular responses to DNA damage^   14 ^. Since the APAF-1gene, TSG, is rarely mutated, promoter hypermethylation appears to be the mechanism underlying its inactivation15. 

In this study, we examined the methylation pattern in APAF-1 promoter region, as well as the expression of APAF-1 in AML Patients. Our analysis of methylation status of APAF-1 gene promoter in patients with AML showed that APAF-1 hypermethylation frequency was 56.4% (57/101).Methylation of the Apaf-1 gene promoter has been demonstrated in AML, CML, and ALL, suggesting that methylation contributes to the inactivation of Apaf-1 expression^   16 ^.

However, in our literature review, no results were found concerning the frequency of APAF-1 gene promoter methylation in AML patients, and thus it was not possible to compare the frequency of this gene in our patients with other studies on AML patients. The results of this study for the first time validated that the hypermethylation of APAF-1 promoter shows a higher prevalence among the AML patients with FLT3 mutations. Moreover, the prevalence of methylated state was higher in females than in males. Liu et al. in a study covering a broad range of patients examined the effect of gender on DNA methylation and interestingly observed that the methylation pattern of approximately 580 recessive genes was significantly different between the female and male subjects^17^. Thus, the gender is likely to affect the methylation status of APAF-1 gene promoter among the AML patients.

Hemimethylated (MU) state was observed in 44.6% (45/101) of patients. This state is associated with partial methylation of promoter regions, assuming that the hemi-methylated status is actually one pair of CpG dinucleotides with one unmethylated cytosine and one 5-hydroxymethylcytosine (5hmC)^18^. In the evaluation of APAF-1 gene expression in AML patients in whom hypermethylation of the promoter of this gene was found, it was observed that APAF-1 expression was reduced compared to normal control samples. The results showed that APAF-1 promoter hypermethylation was closely correlated with the loss of APAF-1mRNA expression, indicating that the function of this gene may be restored following demethylation.

Studies carried out in leukemic cell lines, as well as in few cases of fresh primary leukemic cells, have shown that the leukemic cells express low levels of APAF-1 in a significant proportion of cases and that their pattern of APAF-1 expression is directly correlated with ultraviolet light-induced apoptosis^19^. Studies have indicated that 25% of human leukemic cell lines, as well as 42% of primary AML cells express a low level of APAF-1 mRNA because of methylation silencing in APAF-1gene^   15 ^.

The present study, along with previous reports (20), determines APAF-1 as another target of methylation silencing. The evidence has shown that demethylation treatment can restore the expression of APAF-1 at both mRNA and protein levels, and therefore activate the apoptotic pathway^   16 ^. Furukawa et al. showed the overexpression of Dnmt1-mediated APAF-1 gene methylation that was reversed by demethylation agents^   15 ^.

When the leukemic cells were treated with 5-aza-2’-deoxycytidine, which is a specific inhibitor of DNA methylation, the expression of APAF-1 was restored in leukemic cells^   15 ^. Deregulated expression of APAF-1 in leukemic cells is a highly complex phenomenon. Indeed, other studies observed that APAF-1 mRNA level was not related with APAF-1 protein levels in AML blasts^   16 ^, which suggests the presence of post-transcriptional defects as well as transcriptional defects.

## CONCLUSION

 We have shown that the decreased expression of APAF-1 gene among AML patients could be caused by promoter hypermethylation. The relationship between methylation status of APAF-1 promoter and the expression level of its mRNA transcript with APAF-1 protein level in blood was not studied, which was a limitation of this study. It is also recommended that the mRNA expression level of APAF-1 gene be assessed in all patients regardless of the methylation status of this gene.
